# Cinnamtannin B-1 Promotes Migration of Mesenchymal Stem Cells and Accelerates Wound Healing in Mice

**DOI:** 10.1371/journal.pone.0144166

**Published:** 2015-12-11

**Authors:** Kosuke Fujita, Katsunori Kuge, Noriyasu Ozawa, Shunya Sahara, Kaori Zaiki, Koichi Nakaoji, Kazuhiko Hamada, Yukiko Takenaka, Takao Tanahashi, Katsuto Tamai, Yasufumi Kaneda, Akito Maeda

**Affiliations:** 1 Skin Regeneration, PIAS Collaborative Research, UIC, Osaka University, Suita, Osaka, 565–0871, Japan; 2 Research and Development Division, PIAS Corporation, Kobe, Hyogo, 651–2241, Japan; 3 Organic Chemistry Department, Kobe Pharmaceutical University, Kobe, Hyogo, 658–8558, Japan; 4 Division of Stem Cell Therapy Science, Graduate School of Medicine, Osaka University, Suita, Osaka, 565–0871, Japan; 5 Division of Gene Therapy Science, Graduate School of Medicine, Osaka University, Suita, Osaka, 565–0871, Japan; Children's Hospital Boston/Harvard Medical School, UNITED STATES

## Abstract

Substances that enhance the migration of mesenchymal stem cells to damaged sites have the potential to improve the effectiveness of tissue repair. We previously found that ethanol extracts of *Mallotus philippinensis* bark promoted migration of mesenchymal stem cells and improved wound healing in a mouse model. We also demonstrated that bark extracts contain cinnamtannin B-1, a flavonoid with *in vitro* migratory activity against mesenchymal stem cells. However, the *in vivo* effects of cinnamtannin B-1 on the migration of mesenchymal stem cells and underlying mechanism of this action remain unknown. Therefore, we examined the effects of cinnamtannin B-1 on *in vivo* migration of mesenchymal stem cells and wound healing in mice. In addition, we characterized cinnamtannin B-1-induced migration of mesenchymal stem cells pharmacologically and structurally. The mobilization of endogenous mesenchymal stem cells into the blood circulation was enhanced in cinnamtannin B-1-treated mice as shown by flow cytometric analysis of peripheral blood cells. Whole animal imaging analysis using luciferase-expressing mesenchymal stem cells as a tracer revealed that cinnamtannin B-1 increased the homing of mesenchymal stem cells to wounds and accelerated healing in a diabetic mouse model. Additionally, the cinnamtannin B-1-induced migration of mesenchymal stem cells was pharmacologically susceptible to inhibitors of phosphatidylinositol 3-kinase, phospholipase C, lipoxygenase, and purines. Furthermore, biflavonoids with similar structural features to cinnamtannin B-1 also augmented the migration of mesenchymal stem cells by similar pharmacological mechanisms. These results demonstrate that cinnamtannin B-1 promoted mesenchymal stem cell migration *in vivo* and improved wound healing in mice. Furthermore, the results reveal that cinnamtannin B-1-induced migration of mesenchymal stem cells may be mediated by specific signaling pathways, and the flavonoid skeleton may be relevant to its effects on mesenchymal stem cell migration.

## Introduction

Mesenchymal stem cells (MSCs) have the ability to differentiate into various cell types and secrete proregenerative factors that contribute to tissue repair [1,2]. Several studies have indicated that MSCs migrate to the wound site during the healing process [3,4]. MSCs are thought to migrate from the bone marrow or perivascular regions of the blood vessels to the blood circulation in response to signals released following tissue damage [5]. In addition, recent studies show that the platelet-derived growth factor receptor (PDGFR)-α-positive nonhematopoietic cell population in blood circulation after tissue injury contains ectoderm-derived MSCs [6,7] and accumulates in damaged tissues [8]. Therefore, enhancing the mobilization of endogenous MSCs to wound sites has the potential to improve the healing process [9,10]. The development of methods to enhance the homing of the stem cells to specific tissues is required in cell therapy and, therefore, various approaches have been used in an attempt to achieve this in animal models [11]. Due to the effectiveness of the immunomodulatory capability of the stem cells, use of the mobilization of autologous stem cells as cell therapy has been attempted in chronic metabolic diseases, besides wound healing [12–14]. Therefore, the identification of new materials that enhance the mobilization of resident MSCs or the homing of circulating MSCs in the peripheral blood may improve current therapeutic approaches.

The *Mallotus philippinensis* Muell-Arg (Euphorbiaceae) plant is widely distributed throughout the Southern regions of Asia and is used in traditional medicine [15]. Various parts of the plant are used for treating helminthic infestations, diabetes, and in wound healing. Recently, we demonstrated that ethanol extracts of *M*. *philippinensis* bark (EMPB) promoted MSC migration and wound healing in a mouse model [16]. We found that injection of EMPB into mice promoted the mobilization of endogenous MSCs, by analyzing their number in the blood circulation. We also traced the MSCs expressing firefly luciferase (ffluc) in EMPB-treated nude mice bearing wounds and found that MSC homing to the wound sites was enhanced. Furthermore, we demonstrated that EMPB induced the migration of MSCs more than it did that of other skin cell types and accelerated wound healing in mice. We found that increased epithelialization activity, angiogenesis, granulation tissue formation, and remodeling in the wound healing process might reduce the wound size.

We reported that EMPB contained protocatechuic and salicylic acids, as well as cinnamtannin B-1, which we demonstrated, could be responsible for the main in vitro chemotactic activity of the extract among these other compounds. Cinnamtannin B-1 possesses several phenolic hydroxyl groups and is reported to exhibit antioxidant property, antimicrobial activities, and anti-platelet aggregation [17–19] that may protect damaged tissues in wounds [20]. However, the effects of cinnamtannin B-1 on the migration activity of MSCs in vivo and wound healing in mice remain unclear.

The migration of MSCs is stimulated by cytokines via several signaling pathways [21], which are defined molecularly and pharmacologically. It has been reported that the migration of MSCs was inhibited by the phosphatidylinositol 3-kinase (PI3K) inhibitor LY294002 but not the protein kinase C (PKC) inhibitor Gö6983 [22]. Protein kinases were also reported to regulate the chemokine-directed migration of MSCs [21]. The mechanism of the cinnamtannin B-1-induced migration might likely be mediated by these particular pathways. Therefore, the relationship between the cinnamtannin B-1-induced migration and the signal pathways in MSCs could be characterized using signal pathway-selective inhibitors.

Furthermore, several flavonoids also promote cell migration in various cells and improve wound healing [23,24]. 7-Carboxymethyloxy-3',4',5-trimethoxy flavone promotes gastric epithelial cell migration [23]. Flavones, isoflavones, and flavanones stimulate the keratinocyte migration, and the influence of the flavonoid structure in the migratory activity has been elucidated. [24]. It is still unclear if flavonoids that activate the migration of MSCs besides cinnamtannin B-1 exists, and the influence of the structural features of cinnamtannin B-1 on these effects remain unknown.

Therefore, in this study, we evaluated the effects of cinnamtannin B-1 on the migratory activity of MSCs in vivo and wound healing in mice. In addition, we pharmacologically characterized the possible underlying mechanisms of the cinnamtannin B-1-induced migration of MSCs using target-selective inhibitors in vitro. Furthermore, we examined the structure-activity relationship between cinnamtannin B-1 and induction of MSC migration using the biflavonoids, monomeric flavonoids, and polyphenols, as its related structural units.

## Materials and Methods

### Ethics statement

All animal experimental protocols were approved by the Osaka University Graduate School of Medicine Standing Committee on Animals (J004879-004).

### Chemicals and reagents

Cinnamtannin B-1 was purchased from Enzo Life Sciences, Inc., (New York, NY, USA). The target-verified inhibitors including LOPAC1280 used in the inhibition assay were obtained from Sigma-Aldrich Corp., (St. Louis, MO, USA). The PI3K and PKC inhibitors (LY294002 and Gö6983, respectively) were purchased from EMD Millipore Corp., (Billerica, MA, USA). The phospholipase C (PLC), lipoxygenase (LOX), and purine inhibitors (U-73343, nordihydroguaiaretic acid, and azathioprine, respectively) were obtained from Sigma-Aldrich Corp., (St. Louis, MO, USA).

The biflavonoids used were procyanidins and biflavonoids from *Cupressus* and *Garcinia* [25,26], which are similar to the procyanidins. Procyanidin A2, procyanidin B1, and cupressuflavone were obtained from Extrasynthese (Genay, France). For the monomeric flavonoids and polyphenols, (+)-catechin was purchased from Nagara Science Co., Ltd., (Gifu, Japan). Genistein was purchased from Sigma-Aldrich Corp., (St. Louis, MO, USA). Caffeic and gallic acids were obtained from LKT Laboratories, Inc., (St. Paul, MN, USA).

### Extracts and fractions

Morelloflavone was prepared from the bark of *Garcinia vilersiana* Pierre, collected in Thailand. Briefly, the dried bark of *G*. *vilersiana* Pierre was extracted with hot methanol and concentrated under reduced pressure to obtain a residue. A sample of the methanol extract (50.0 g) was dissolved in water (H_2_O) and extracted successively with chloroform (CHCl_3_) and n-butanol to obtain three fractions with yields of 0.9, 37.0, and 120 g (CHCl_3_, n-butanol, and H_2_O, respectively). The n-butanol fraction was chromatographed on a Wakosil C18 gel flash column (Wako) and eluted with a gradient of H_2_O:methanol from 100:0 to 0:100 (v/v) to obtain 20 fractions. A sample of the fractions (500 mg, 50% methanol eluent, 7.04 g) was further purified using preparative thin-layer chromatography (TLC) and a mobile phase consisting of benzene:ethyl acetate:ethanol (6:2:1) to obtain (+)-morelloflavone (yield, 243 mg). Samples were identified by comparing their physical and spectral data with the corresponding previously reported values [27]. TLC was performed on precoated Kieselgel 60F_254_ plates (Merck, Darmstadt, Germany). All organic solvents were purchased from Nacalai Tesque, Inc., (Kyoto, Japan).

### Cell culture

The mouse MSC cell line derived from female C3H/He bone marrow, KUM6 cells [28] was provided by the RIKEN BRC (Ibaraki, Japan) through the National Bio-Resource Project of the Ministry of Education, Culture, Sports, Science, and Technology (MEXT), Japan and maintained in Poweredby 10 culture medium (GlycoTechnica, Yokohama, Japan).

### Animals

Male C57BL/6NJcl, female BALB/cAJcl-nu/nu, and female C57BLKS/Jlar-+Lepr^*db*^/+Lepr^*db*^ mice (11-, 7-, and 9-week-old, respectively) were obtained from Clea Japan (Tokyo, Japan). Mice were housed in a sterile animal facility with the temperature controlled at 23 ± 1.5°C and 50% relative humidity under a 12 h light/dark cycle and free access to standard laboratory diet and water.

### *In vivo* MSC mobilization

Six male C57BL/6 mice each from the control and cinnamtannin B-1-treated groups were injected with 0.4 mL phosphate-buffered saline (PBS) or cinnamtannin B-1 solution (2.0 mg/kg via the vena caudalis), respectively. The experiments were performed five times. After 1 h, approximately 1 mL samples of peripheral blood were collected, and nucleated cells were separated using Ficoll gradient centrifugation (final density, 1.077 g/m). To detect the endogenous MSCs with the lineage-negative, PDGFRα+, and Sca-1+ (Lin-/PDGFRα+/Sca-1+) cells were stained with allophycocyanin (APC)-conjugated anti-PDGFRα (eBioscience, San Diego, CA, USA), PE-conjugated anti-Sca-1 (eBioscience), or V450 Mouse Lineage Antibody Cocktail (BD Biosciences, San Jose, USA) diluted in PBS and fixed with 1% paraformaldehyde. Flow cytometry analysis was performed using a FACSCanto II flow cytometer (BD Biosciences) using FACSDiva software. The percentage of peripheral blood mononuclear fraction cells that targeted the Lin-/PDGFRα+/Sca-1+ in the control and cinnamtannin B-1 groups was compared.

### *In vivo* MSC homing assay

Nude mice were used to detect the subcutaneous bioluminescence induced by luciferase [29]. Briefly, female BALB/c-nu/nu mice were swabbed clean with 70% ethanol, and full-thickness wounds were created using the 8-mm punch biopsy on their backs under anesthesia with isoflurane. The φ6 × 2 mm, pore size 200–400 μm sterile collagen sponges (AteloCell, Koken Co., Ltd., Tokyo, Japan) containing 8.0 μg cinnamtannin B-1 suspended in 40 μL PBS or PBS alone (control group) were implanted as sustained release agents in the wounds, which were immediately covered with Tegaderm (Sumitomo 3M, Tokyo, Japan). During surgery, mice were anesthetized with isoflurane. After the wound induction, the condition of the mice was monitored every day. A total of 6–10 samples per group were used, and the experiment was performed in duplicate.

On day 4 post surgery, the mice were injected via the tail vein under anesthesia with 2.5 × 10^5^ KUM6 cells transiently transfected with an expression vector for firefly luciferase (ffluc) pGL4.50 (Promega, Madison, WI, USA), using the Neon^TM^ Transfection system (Thermo Fisher Scientific, Waltham, MA, USA) according to the manufacturer’s recommendations. To confirm the transfection efficiency in every experiment, the levels of luciferase expression in the transfected KUM6 cells were measured separately in vitro, by adding the reporter substrate D-luciferin (PerkinElmer, Inc., Waltham, MA, USA) 32 h after transfection using an IVIS imaging system (PerkinElmer, Inc., Waltham, MA, USA). To detect the luminescence activity of the expressed luciferase in MSCs in vivo, the D-luciferin (150 mg/mL) was injected into the mice intraperitoneally, and measurements were performed 20 min later. During measurement, mice were anesthetized with isoflurane. The bioluminescence was measured on day 2 and 5 after injections using an IVIS imaging system. At the end of the experiment, the mice were euthanized by deep anesthesia with isoflurane.

The luciferase-expressing KUM6 cells that accumulated in the wound were quantified based on the intensity of the emission, and the analysis was carried out using the Living Image In Vivo Imaging software (PerkinElmer, Inc., Waltham, MA, USA).

## Wound healing model

Genetically diabetic C57BLKS/Jlar-+Lepr^*db*^/+Lepr^*db*^ mice were used in the wound healing model because their slow wound healing rate is reported to be effective for drug efficacy evaluation [30]. The hair was removed from the backs of the mice using a depilatory under anesthesia, their backs were wiped with 70% ethanol, and then 1.5 cm full-thickness wounds were created using scissors. During surgery, mice were anesthetized with isoflurane. After the wound induction, the condition of mice was monitored every day. Cinnamtannin B-1 suspended in PBS (group I and II, 1.2 and 2.4 μg/wound, respectively), or PBS alone (control) was topically applied three times per week, and the wound areas and surface condition were measured from the acquired photographs after cinnamtannin B-1 treatment. During manipulations, mice were anesthetized with isoflurane. The exposed wound area was immediately covered with Tegaderm following treatment with cinnamtannin B-1. Ten samples were analyzed per group, and the experiment was performed in triplicate. At the end of the experiment, the mice were euthanized by deep anesthesia with isoflurane. The acquired images of the wound area were measured using the ImageJ software (National Institutes for Health, NIH, Bethesda, MD, USA, NIH-http://rsb.info.nih.gov/ij/), and the wound area percentage (%) was calculated relative to the total area on day 0, which was denoted as 100%.

Wound tissue samples were harvested on day 14, fixed with 10% neutral-buffered formalin, embedded in paraffin wax, and serial 4-μm sections were cut from the central portion of the wound tissue. The sections were then evaluated using α-smooth muscle actin (α-SMA) immunostaining with anti-α-SMA monoclonal antibodies (Agilent Technologies, Santa Clara, CA, USA), as well as hematoxylin and eosin (H&E) and Masson Trichrome staining. Micrographs were obtained using a light microscope IX-71 (Olympus, Tokyo, Japan). Images of the entire wound tissue created from digital images acquired using a biological microscope were analyzed using the CellSens imaging software (Olympus). The number of α-SMA-positive vessels in the dermis of the entire scar area was measured. The granulation tissue area was calculated as the blue-stained portion of the wound tissue after capturing the image of the Masson Trichrome-stained specimens. Six specimens were used per group for each analysis.

### Cell migration assay

The migration of KUM6 cells was assayed using a modified Boyden chambers as described previously [31]. In brief, the compounds solution in 28.0 μL serum-free Dulbecco’s modified Eagle’s medium (DMEM, Wako, Osaka, Japan) and 3 × 10^5^ cells/mL suspended in 50 μL Poweredby 10 medium were added to the lower and upper chambers, respectively. After a 4 h incubation, the membrane inserted between both chambers was removed, and the cells on the lower surface of the membrane were stained with Diff-Quick (Sysmex, Kobe, Japan). The number of cells that migrated was analyzed using a CanoScan 8800F scanner (Canon, Tokyo, Japan) for the detection of stained cells.

For the pharmacological assay with the inhibitors, the cinnamtannin B-1-treated KUM6 cells were incubated with 10–25 μM LY294002, 0.625–2.5 μM U-73343, 12.5–50 nM Gö6983, 6.25–25 μM nordihydroguaiaretic acid, or 50–100 μM azathioprine. The migration of cells incubated with serum-free DMEM only was measured as the control. The migration index of the inhibitors was calculated after normalization to the value of cinnamtannin B-1 to evaluate the chemotaxis. The migration index at 20 μg/mL cinnamtannin B-1 was set as 100%. Four samples were evaluated per group, and the experiment was performed in duplicate.

For the structure-activity relationship assay, we used the flavonoid and polyphenol as homologous units to cinnamtannin B-1. The active compounds were used at a concentration range of 0.16–20 μg/mL based on the reaction concentration of cinnamtannin B-1. Cupressuflavone, monomeric flavonoids, and polyphenols were used at 0.2–200 μg/mL because we sought to determine if they were active over a wide concentration range. The migration of cells incubated with the serum-free DMEM alone was measured as the control. The migration index was calculated by first normalizing the data to the value obtained with PDGF-BB to evaluate the chemotaxis. The migration index at 20 ng/mL PDGF-BB in DMEM was set as 100%. Four samples were analyzed per group, and the experiment was performed in duplicate.

For the pharmacological assay, we measured the migration of biflavonoid-treated KUM6 cells incubated with the inhibitors LY294002 (25 μM), Gö6983 (50 nM), U-73343 (1 μM), nordihydroguaiaretic acid (25 μM), or azathioprine (100 μM). As a control, the migration of cells incubated in serum-free DMEM only was measured. The migration index of the inhibitors was calculated after normalizing the data to the value of biflavonoid to evaluate the chemotaxis. The migration index of biflavonoid at 20 μg/mL was set at 100%. Four samples were analyzed per group, and the experiment was performed in duplicate.

### Cell proliferation assay

The growth rate of KUM6 cells was measured using the 3-(4,5-dimethylthiazol-2-yl)-5-(3-carboxymethoxyphenyl)-2-(4-sulfophenyl)-2H-tetrazolium (MTS) assay as described previously [31]. In brief, KUM6 cells within five passages were suspended in DMEM supplemented with 10% fetal bovine serum (FBS, Biosera, France) and seeded at a density of 2 × 10^3^ cells/well in a 96-well plate. After 24 h, the medium was changed to DMEM supplemented with 1% FBS for 24 h, and then the cells were treated for 24 h with the same concentration of compounds used in the cell migration assay. The CellTiter 96 One Solution reagent (Promega, Madison, WI, USA) was added to each well and the absorbance was measured at 490 nm. As a control, the proliferation activity of cells incubated with 1% FBS-containing DMEM only was measured. The relative number of cells was calculated by normalizing the data to the control value, which was set as 100%. Six samples were analyzed per group, and the experiment was performed in duplicate.

### Statistical analysis

Results are expressed as means ± standard error (SE). The analysis of variance (ANOVA) and Dunnett’s test were used to determine the significant differences between the control and other groups. For the proliferation assay with caffeic acid, the data were analyzed using the Steel’s test followed by Kruskal-Wallis test.

## Results

### Enhancement of MSC migration by cinnamtannin B-1 *in vivo*

**Fig 1:**
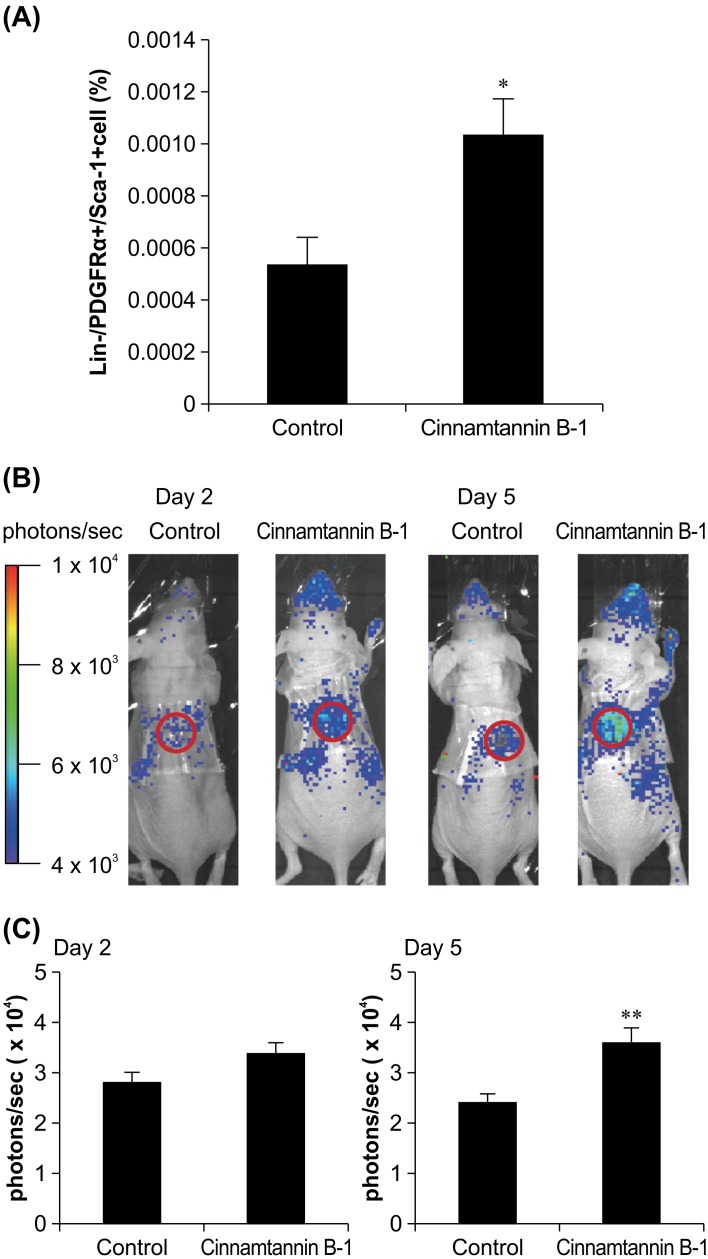
Effects of cinnamtannin B-1 on migration of mouse mesenchymal stem cells (MSCs). (A) Increase in population of Lin−/PDGFRα+/Sca-1+ peripheral blood mononuclear cells of mice after systemic administration of cinnamtannin B-1, n = 6 per group. ^*^ *P* < 0.05 vs. control. (B) KUM6 cells localized in wound healing models; ffLuc-expressing KUM6 cells were injected 4 days after surgical incision (day 1). Images of a representative animal are shown on day 2 and 5 after ffLuc-KUM6 cell injection. (C) Quantification of photon counts at wound site on day 2 and 5 (n = 8–9 and 6–7, respectively); ^**^ *P* < 0.01 vs. control.

We evaluated the effects of cinnamtannin B-1 on the in vivo migration of endogenous MSCs to the blood circulation as well as their homing to wounds. We analyzed the number of MSCs in the blood circulation after injecting cinnamtannin B-1 into mice. Flow cytometric analysis revealed that the population of lineage-negative, PDGFRα+, and Sca-1+ (Lin-/PDGFRα+/Sca-1+) cells was increased in cinnamtannin B-1-treated mice compared with the control (Fig 1A). Therefore, the results indicate that cinnamtannin B-1 enhanced the mobilization of endogenous MSCs to the blood circulation.

We further examined whether cinnamtannin B-1 also enhanced the homing of MSCs to wounded sites. We injected MSCs transiently expressing ffluc as a tracer into nude mice bearing dorsal wounds treated with cinnamtannin B-1 and used bioluminescence to perform whole-animal imaging. The results showed that ffluc-expressing MSCs were increased in the region of the wound (Fig 1B). Quantification of the photon counts at the wound site revealed a 1.5-fold higher increase with cinnamtannin B-1 treatment than with the untreated control at day 5 (Fig 1C). The results indicate that cinnamtannin B-1 also enhanced the homing of MSCs from the blood circulation to wounds and promoted the in vivo mobilization of resident and homing of circulating MSCs.

### Effects of cinnamtannin B-1 on wound healing in mice

**Fig 2:**
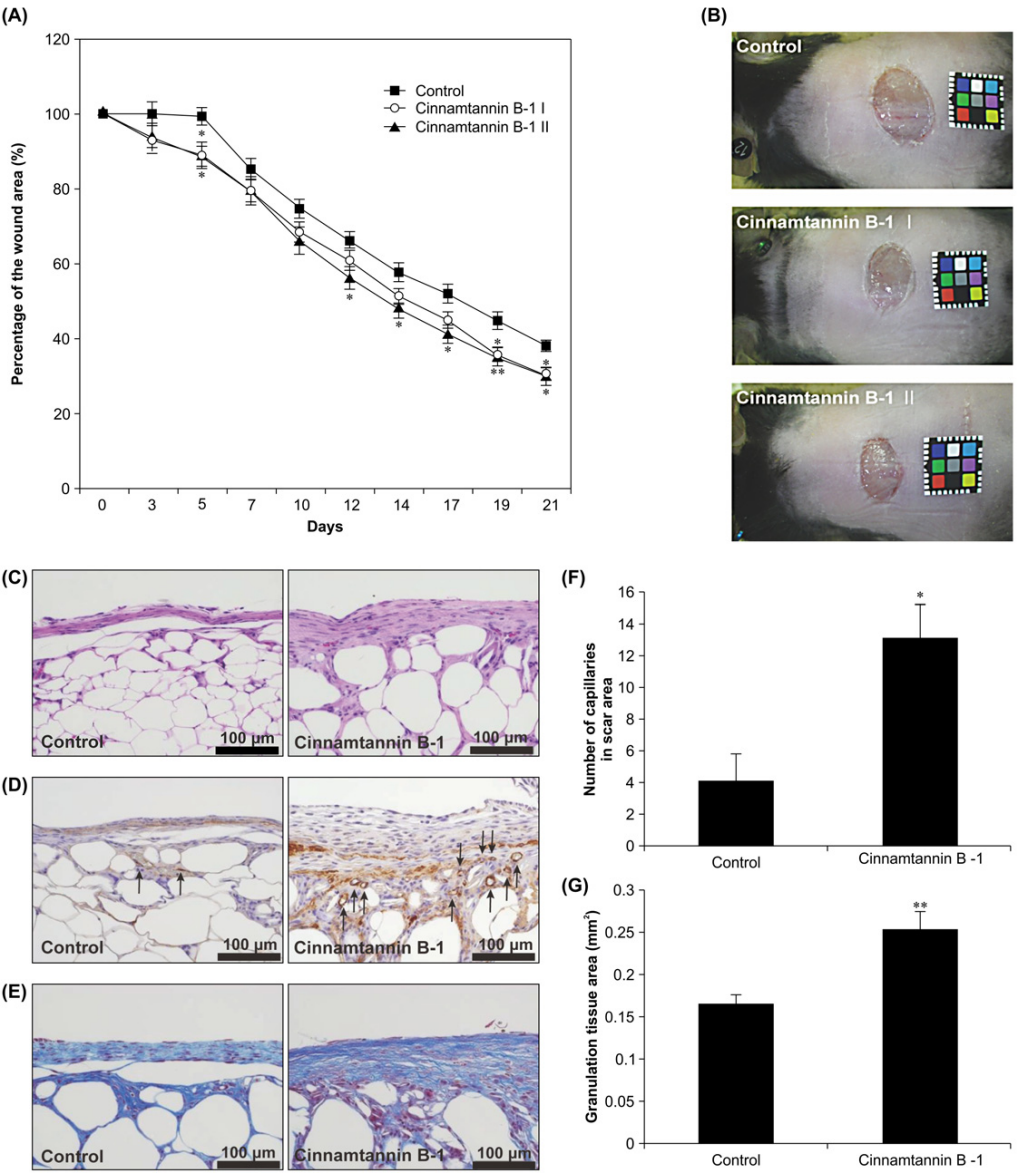
Effects of cinnamtannin B-1 in a diabetic mouse wound healing model. Topical application of cinnamtannin B-1 or phosphate-buffered saline (PBS, control) was repeated three times per week. (A) Time course of quantification of wound size. Percentage (%) of wound area was calculated as wound area at different times/wound area on day 0 × 100; n = 10 in each group, ^*^ *P* < 0.05 and ^**^ *P* < 0.01 vs. control. (B) Representative images of wounds on day 19. (C) Hematoxylin and eosin (H&E) staining of specimens on day 14 (200× magnification). (D) α-SMA immunostaining (200× magnification). The capillaries are indicated by arrows. (E) Masson Trichrome staining (200× magnification). (F) Number of capillaries in scar area was counted; n = 6, ^*^ *P* < 0.05 vs. control. (G) Granulation tissue area (mm^2^) was calculated as blue stained portion in wound; n = 6, ^**^ *P* < 0.01 vs. control.

The effects of cinnamtannin B-1 on wound healing were analyzed by determining the reduction in the wound size in a diabetic mouse model. Based on the in vitro activity, two doses of cinnamtannin B-1 were used. Cinnamtannin B-1 II (2.4 μg/wound)-treated mice indicated a significant reduction in the wound area on day 5, 12, 14, 17, 19, and 21 compared to the control (Fig 2A and 2B). A reduction in the wound area on day 5, 19, and 21 was also observed in the cinnamtannin B-1 I (1.2 μg/wound)-treated mice.

We further examined the effects of cinnamtannin B-1 on tissue regeneration in wounds. Randomly selected photographs of wound tissue on day 14 following treatment of mice with PBS (control) or cinnamtannin B-1 II are shown in Fig 2C–2E. The epithelialization and formation of granulation tissue were highly developed in cinnamtannin B-1 II-treated mice but not the controls. Furthermore, the granulation tissue of the cinnamtannin B-1 II-treated mice was more solid that of the control mice. These data imply that cinnamtannin B-1 improved wound healing by facilitating tissue regeneration. Next, we compared angiogenesis in the wound area, by evaluating α-SMA staining of the entire granulation area in mice treated with PBS (control) or cinnamtannin B-1 II. Compared to the control mice, the wound area of cinnamtannin B-1 II-treated mice exhibited a significant increase in capillaries (arrows, Fig 2D and 2F). Masson Trichrome staining also showed a higher increase in granulation tissue in the cinnamtannin B-1 II-treated than in untreated control mice (Fig 2E and 2G). These results indicate that cinnamtannin B-1 enhanced the wound healing in mice. Furthermore, the healing observed in the tissue specimens demonstrates that cinnamtannin B-1 may accelerate tissue regeneration during the wound healing process. The histopathological characteristics of the wound healing following cinnamtannin B-1 treatment were similar to those observed with EMPB treatment in previous studies [16]. Therefore, these results suggest that the characteristics of tissue regeneration resemble MSC-induced tissue repair.

### Pharmacological characterization of cinnamtannin B-1-induced migration of MSCs

**Fig 3:**
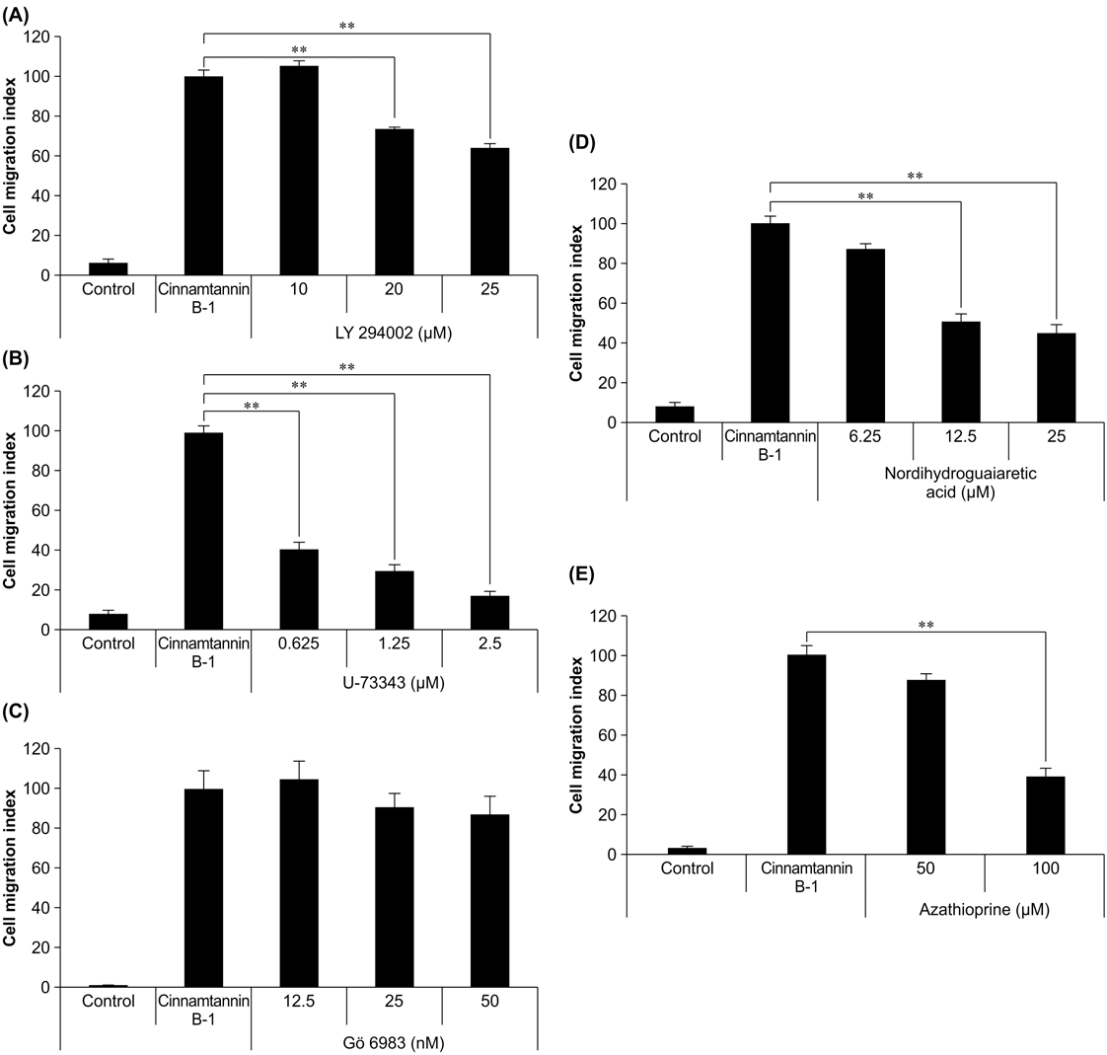
Effects of specific inhibitors on cinnamtannin B-1-induced migration of KUM6 cells. (A) Phosphatidylinositol 3-kinase (PI3K) inhibitor, LY294002. (B) Phospholipase C (PLC) inhibitor, U-73343. (C) Protein kinase C (PKC) inhibitor, Gö6983. (D) Lipoxygenase (LOX) inhibitor, nordihydroguaiaretic acid. (E) Purine inhibitor, azathioprine. Values are mean ± SE while migration indices are normalized to that of cinnamtannin B-1-treated cells; n = 4/group, ^**^ *P* < 0.01 vs. control.

To pharmacologically investigate the mechanism underlying the cinnamtannin B-1-induced MSC migration, the effects of the specific inhibitors were analyzed using migration assay with the MSC cell line, KUM6. We examined the effects of the Library of Pharmacologically Active Compounds (LOPAC1280), which contains a set of 1280 pieces of the target-verified inhibitors, and consequently found selective drugs. The inhibitors including LY294002 (PI3K), U-73343 (PLC), nordihydroguaiaretic acid (LOX), and azathioprine (purine) inhibited the cinnamtannin B-1-induced MSC migration (Fig 3), whereas the PKC inhibitor Gö6983 (negative control) did not inhibit the migration activity. Therefore, these results suggest that specific signaling pathways may be involved in the cinnamtannin B-1-induced migration of MSCs.

### Effects of biflavonoids, monomeric flavonoids, and polyphenols on migration and proliferation of MSCs

**Fig 4:**
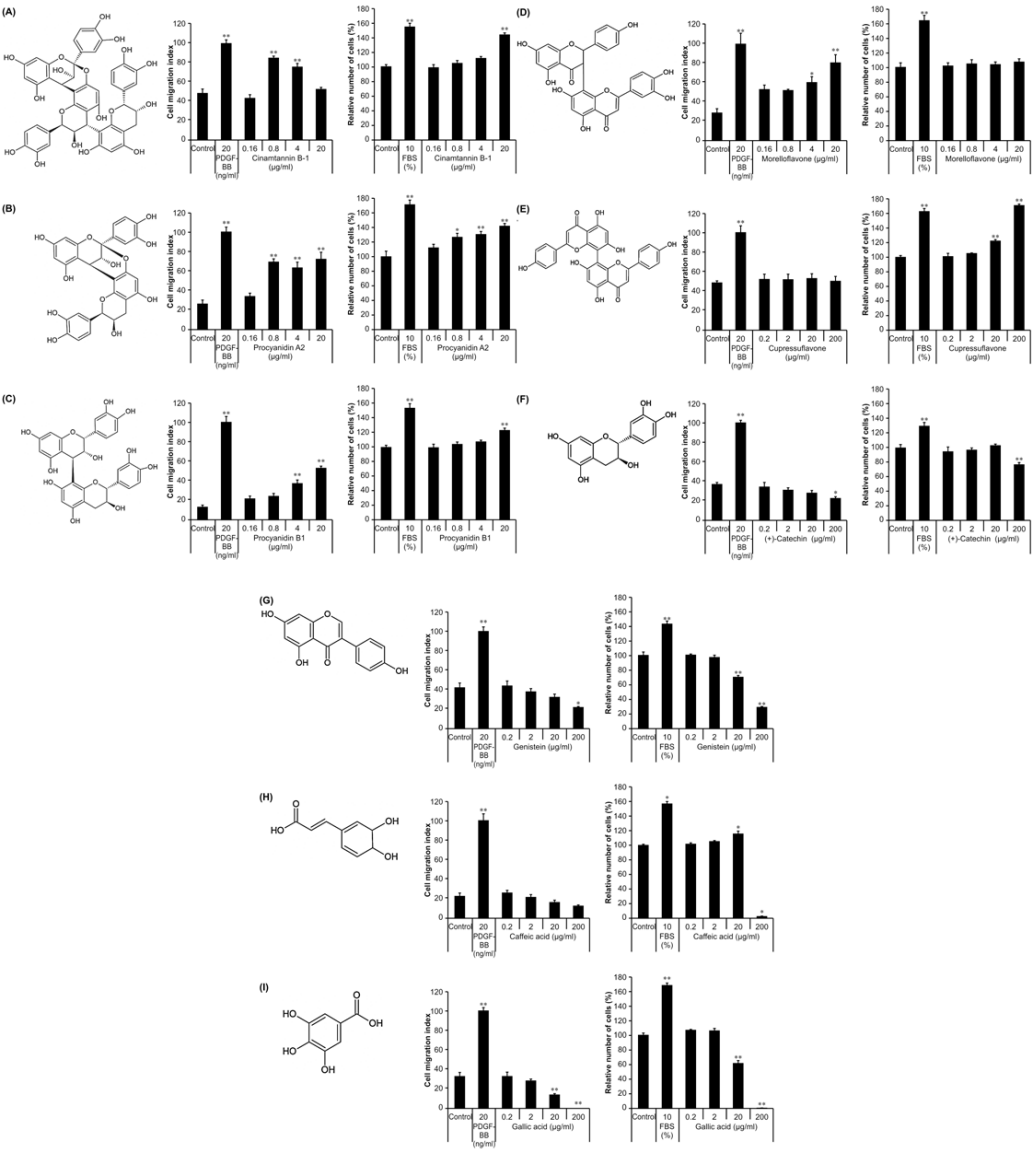
Effects of various flavonoids and polyphenols on KUM6 cells. (A) Structure of cinnamtannin B-1 and MSC migration and proliferation after stimulation with cinnamtannin B-1, (B) procyanidin A2, (C) procyanidin B1, (D) morelloflavone, (E) cupressuflavone, (F) (+)-catechin, (G) genistein, (H) caffeic acid, and (I) gallic acid. Cell migration assay values are means ± SE and indices were normalized to platelet-derived growth factor (PDGF)-BB-treated cells; n = 4/group, ^*^ *P* < 0.05 and ^**^ *P* < 0.01 vs. control. Cell proliferation assay values are mean ± SE percentages normalized to that of untreated controls; n = 6/group, ^*^ *P* < 0.05 and ^**^ *P* < 0.01 vs. control.

We examined whether the structural features of cinnamtannin B-1 affected the migration of MSCs by evaluating the effects of biflavonoids, monomeric flavonoids, and polyphenol as structural units of cinnamtannin B-1 (Fig 4A). We determined their effects in the migration assay using KUM6 cells and a proliferation assay to verify toxicity.

In the experiment with the biflavonoids, treatment with 0.8–20 and 4–20 μg/mL procyanidin A2 and procyanidin B1, respectively increased the migration of KUM6 cells compared with the negative control (Fig 4B and 4C), indicating that the biflavonoids enhanced the migration of KUM6 cells. The proliferation of KUM6 cells was also enhanced following 24 h exposure to procyanidin A2 (0.8–20 μg/mL, Fig 4B) and procyanidin B1 (20 μg/mL, Fig 4C).

To determine the influence of other biflavonoid, we examined the effects of the structurally different but slightly similar morelloflavone, which was also promoted the migration of KUM6 cells at concentrations of 4–20 μg/mL (Fig 4D). However, it did not affect proliferation of KUM6 cells. In contrast, cupressuflavone showed no significant effect on the migration of KUM6 cells but increased their proliferation at a high concentration (Fig 4E).

Additionally, the application of the monomeric flavonoids catechin and genistein, as well as the polyphenols caffeic and gallic acid, also did not alter the migration of MSCs, but a high concentration inhibited the proliferation of KUM6 cells (Fig 4F–4I). These results indicate that there is a degree of structure-activity relationship between the biflavonoids and the MSC migration activity.

### Pharmacological characterization of biflavonoid-induced migration of MSCs

**Fig 5:**
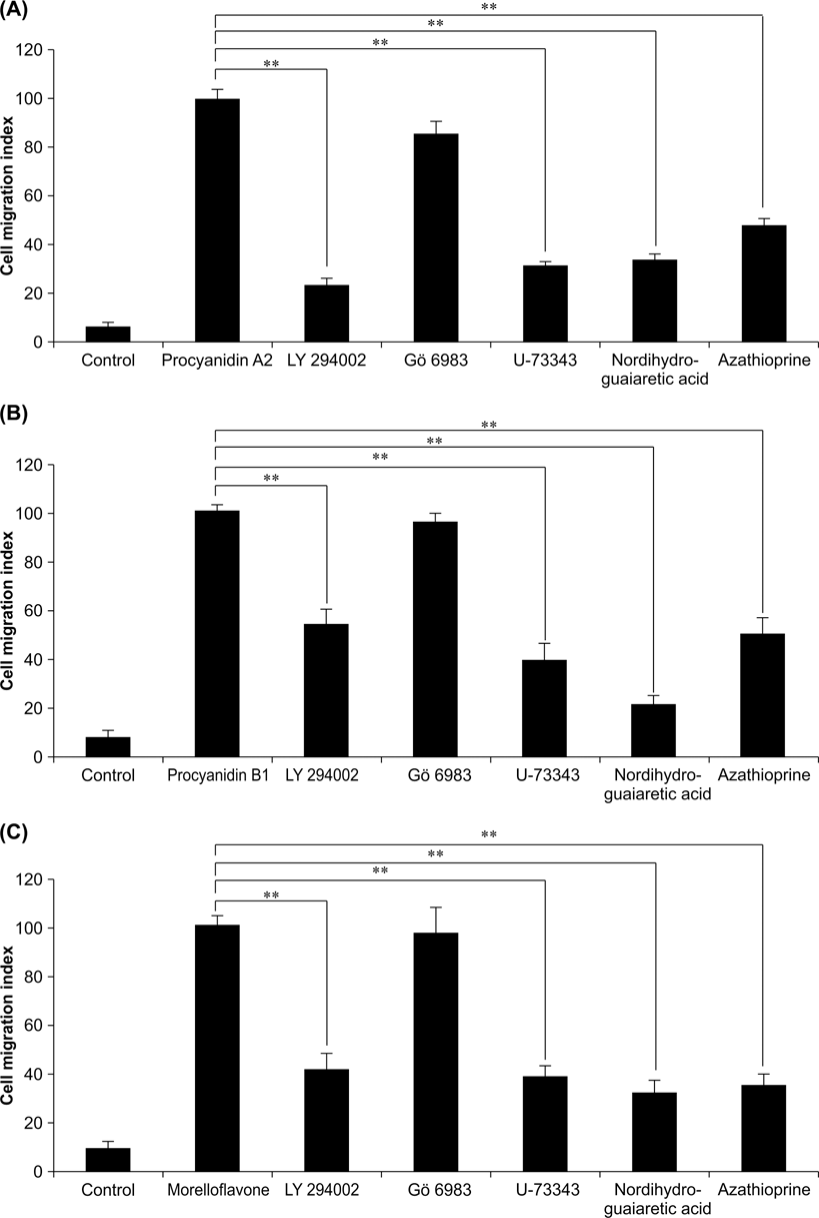
Effects of specific inhibitors on biflavonoid-induced migration of KUM6 cells. (A) Procyanidin A2, (B) procyanidin B1, and (C) morelloflavone. Inhibitors were used at 25 μM, 50 nM, 1 μM, 25 μM, and 100 μM for LY294002, Gö6983, U-73343, nordihydroguaiaretic acid, and azathioprine, respectively. Values are mean ± SE of migration indices normalized to that of biflavonoid-treated cells; n = 4/group, ^**^ *P* < 0.01 vs. control.

Next, we used specific signaling pathway inhibitors to pharmacologically investigate the mechanism of the biflavonoid-induced MSC migration activity using the migration assay. The specific inhibitors of PI3K (LY294002), PLC (U-73343), LOX (nordihydroguaiaretic acid), and purine (azathioprine) but not of PKC (Gö6983) inhibited the biflavonoid-induced migration of MSCs at a reasonable inhibitory concentration similar to that of cinnamtannin B-1 (Fig 5). These results indicate that the pharmacological profiles of the biflavonoid- and cinnamtannin B-1-induced MSC migration are similar and are likely mediated by common mechanisms. Taken together with the structural analysis, these results demonstrate that the biflavonoid portion of cinnamtannin B-1 might be responsible for the MSC migratory actions.

## Discussion

We previously reported that EMPB enhances the migration of MSCs and mobilizes them to the wound site, further promoting wound healing [16]. The MSC migration activity of EMPB may mainly be attributed to cinnamtannin B-1, which is one of its major constituents. In the present study, we analyzed the effects of cinnamtannin B-1 on MSC migration in vivo and demonstrated that it mobilized the MSCs from the bone marrow to the blood from where they moved to accumulate at the wound site. We also found that topical application of cinnamtannin B-1 promoted healing of the wound sites. Histopathological analysis following cinnamtannin B-1 treatment showed that angiogenesis, granulation tissue formation, and remodeling were accelerated during the wound healing process, similar to the effects previously observed with EMPB [16]. MSCs reportedly promote wound closure, vascularization, and thickening of granulation tissue [4]. MSCs also mediate immunomodulatory effects such as the secretion various cytokines having proregenerative activities for damaged cells and the inhibition of inflammation [2,14]. Interestingly, our results suggest that cinnamtannin B-1 may promote tissue regeneration during the wound healing process and that the healing features of the wound sites might be attributable to the MSC-induced tissue repair. Since excessive inflammation in the surface of the wound and the disturbance of the skin layer structure of wound tissues were also not observed in the treatment of cinnamtannin B-1, we assumed it may be attributed to the anti-inflammatory effect of MSCs. Cinnamtannin B-1 has been reported to exhibit antioxidant and antimicrobial activities, as well as antiplatelet aggregation [17–19]. These activities may have protective effects on damaged tissues and the microenvironment of wounds. Therefore, the effects of cinnamtannin B-1 may correspond to those of EMPB. However, it is important to note that other components of the EMPB might also contribute to this effect and exhibit synergism with cinnamtannin B-1 since protocatechuic acid, which is a component of EMPB, has also been shown to promote the migration of adipose tissue-derived stromal cells in vitro [32].

In addition, we demonstrated that the specific inhibitors of PI3K, PLC, LOX, and purine (LY294002, U-73343, nordihydroguaiaretic acid, and azathioprine, respectively) suppressed the cinnamtannin B-1-induced MSC migration activity. Inhibitors of PI3K and PLC activity have been reported to inhibit the MSC migration activity [21,33]. However, the PKC inhibitor Gö6983 did not alter the MSC migration [22]. We observed that cinnamtannin B-1 stimulates the phosphorylation of p70S6K (S1 Fig). The protein p70S6K is a downstream effector of the PI3K pathway and has been reported to promote cell migration [23,34]. Therefore, the suppression of PI3K pathway may affect MSC migration via actions on p70S6K. Furthermore, we also considered the involvement of LOX activity in the migration of MSC, since the phenotype of LOX-deficient mice indicates an attenuation of the accumulation of migrated MSC at their wound sites [35]. Although the relationship between MSC migration and purine activity is not entirely clear, it has also been reported that azathioprine influences MSC migration via an actin-based cytoskeletal function [36]. Our findings demonstrate that specific signaling pathways may be involved in the cinnamtannin B-1-induced MSC migration. However, since the phenolic hydroxyl group of cinnamtannin B-1 binds nonspecifically to numerous proteins in vitro, we could not identify the cinnamtannin B-1-interacting protein using affinity binding directly. Future studies are planned to further elucidate the specific molecular mechanisms of each specific signaling pathway shown to mediate the cinnamtannin B-1-induced migration of MSCs.

Our structural analysis revealed a structure-specific biflavonoids-induced MSC migration activity. Cinnamtannin B-1 is a trimeric flavonoid, composed of epicatechin-(2β→7, 4β→8)-epicatechin-(4β→8)-epicatechin [37]. It has internal binding from the C-ring to the D-ring and from the F-ring to the G-ring in the skeletal structure of the flavonoid. The structure of procyanidin A2 is epicatechin-(2β→7,4β→8)-epicatechin, and that of procyanidin B1 is epicatechin-(4β→8)-catechin [37]. Therefore, they are structurally similar to part of the cinnamtannin B-1 molecule. In addition, the flavanone-(3→8ʹʹ)-flavone-type biflavonoid morelloflavone, which is structurally analogous to the procyanidin molecule, also showed MSC migration activity. However, the (8→8ʹʹ)-biflavone-type biflavonoid cupressuflavone [26] did not exhibit any effect on MSC migration. Cinnamtannin B-1 and the biflavonoids with MSC migration activity all have the internal binding from the C-ring to the D-ring in the skeleton structure of flavonoid and, therefore, these structures might be critically involved in the interaction with the signal molecules. Furthermore, the MSC migration activity of the biflavonoids was inhibited by the same inhibitors of the signaling pathways suspected to mediate the cinnamtannin B-1-induced MSC migration activity. These results suggest that the MSC migration induced by the biflavonoids and cinnamtannin B-1 may be mediated via common mechanisms. Therefore, our results suggest that flavonoids with similar internal binding modes with the skeleton of cinnamtannin B-1 may also possess MSC migration activity. However, the number and types of flavonoids used as samples in this study were limited. Although the migratory cell effect of flavonoids has been reported with several monomeric flavonoids shown to induce keratinocyte migration [38], the monomeric flavonoids did not exhibit MSC migration activity. Since the EMPB containing cinnamtannin B-1 also did not exhibit keratinocyte migration activity [16], the mechanism mediating the effects of cinnamtannin B-1 might be different from that of the flavonoid-induced keratinocyte migration.

MSCs have the ability to differentiate into various cell lineages [1] as well as secrete regulators of host defense and tissue repair [10]. Recently, numerous clinical studies of MSCs have been conducted [39]. Methods focused on developing ways to control the kinetics of MSCs during and following transplantation would be extremely useful for various applications, such as tissue regeneration in the organs and protection from tissue damage. Cinnamtannin B-1 and the structure-specific biflavonoids with MSC migration activity could act as MSC invitation analogs, similar to the action of cytokines. Therefore, compounds, which control the migration of MSCs could be useful tools in regenerative medicine. Although several benefits of cinnamtannin B-1 such as the in vitro anti-apoptotic effect and anti-platelet aggregation have been reported [17,18], further studies are required to confirm the safety of these compounds, especially concerning possible adverse events such as the side-effects in organs or irregular angiogenesis.

In conclusion, our results demonstrate that cinnamtannin B-1 promoted MSC migration in vivo and accelerate wound healing in mice. In addition, the cinnamtannin B-1-induced migration of MSCs may be mediated by specific signaling pathways. Furthermore, the structural features of flavonoid skeleton of cinnamtannin B-1 may be critical to their effects on the migratory activity of MSCs.

## Supporting Information

S1 Fig Phosphorylation of p70S6K by cinnamtannin B-1. (DOCX)
