# Supplementary material for: Cinnamtannin B-1 Promotes Migration of Mesenchymal Stem Cells and Accelerates Wound Healing in Mice
Source: PLoS One. 2015 Dec 11;10(12):e0144166. doi: 10.1371/journal.pone.0144166 (PMC4686113; doi:10.1371/journal.pone.0144166)
Supplement: S1 Fig — (DOCX) [file pone.0144166.s001.docx]

**Supporting Information**

**S1 Fig. Phosphorylation of p70S6K by cinnamtannin B-1**

Western blot analysis: KUM6 cells were seeded at a density of 1.2 × 10^5^ cells/well in 6-well plates. After 24 h, the cells were treated for 1 h with LY294002 (25 μM) and Poweredby 10 medium was used as a control. After incubation, the cells were treated for 5 min with cinnamtannin B-1 (4 μg/mL) and Poweredby 10 medium was used as a control. Cells were washed with PBS, lysed in 2× sample buffer (Bio-Rad, Hercules, CA, USA), and lysates were analyzed using western blotting. Total lysates were separated using sodium dodecyl sulfate-polyacrylamide gel electrophoresis (SDS-PAGE) under denaturing conditions and transferred to a polyvinylidene fluoride (PVDF) membrane. The membrane was incubated with primary antibodies against phospho-p70S6K (T389, 1:200, 9234S, Cell Signaling Technology, Danvers, MA, USA). Equal loading of samples was verified using β-actin immunoblotting with monoclonal antibody (1:2000, 3700S, Cell Signaling Technology). Then, the membrane was incubated with anti-rabbit (1:1000, 7074S, Cell Signaling Technology) or anti-mouse horseradish peroxidase-conjugated secondary antibody (1:2000, 7076S, Cell Signaling Technology), and developed with the Enhanced Chemiluminescence (ECL) Prime (GE Healthcare UK Ltd., Amersham Place, Little Chalfont, Buckinghamshire HP7 9NA, England). The resulting chemiluminescence was detected with the Chemidoc system (Bio-Rad, Hercules, CA, USA).
